# Experiences of Awe and Gratitude and Related Triggers Among Religious Brothers and Sisters: Findings from a Cross-Sectional Study in Germany

**DOI:** 10.1007/s10943-023-01983-5

**Published:** 2024-01-19

**Authors:** Arndt Büssing, Michael Weit, Klaus Baumann

**Affiliations:** 1 Professorship Quality of Life, Spirituality and Coping, Faculty of Health, Witten/Herdecke University, Gerhard-Kienle-Weg 4, 59313 Herdecke, Germany; 2 IUNCTUS - Competence Center for Christian Spirituality, Philosophical-Theological Academy, Münster, Germany; 3 Caritas Science and Christian Social Work, Faculty of Theology, Albert-Ludwig-University, 79085 Freiburg, Germany

**Keywords:** Awe perception, Triggers, Religious brothers and sisters, Psychological wellbeing, Nature, Religious practices, Connectedness

## Abstract

A cross-sectional survey among religious brothers and sisters (n = 250) with their specific lifestyle and related spiritual practices stated moments of awe perceptions. They responded to both the Awe/Gratitude scale and to free text fields to substantiate their quantitative responses. Qualitative content analysis of their free text responses resulted in six main categories of awe triggers: (1) *Nature*, (2) *Special Moments*, (3) *Transcendence Perceptions*, (4) *Religious practices*, (5) *Distinct People*, and (6) *Aesthetics, Art and Culture*. Awe perceptions can be an immediate feeling and the outcome of a process of reflection in response to admiration, inspiration, and elevation. As these perceptions are related to psychological well-being and prosocial behaviors, their training can generate positive effects on quality of life.

## Introduction

Pausing in specific situations with feelings of wondering awe (Keltner, 2023) and subsequent feelings of gratitude is a perceptive aspect of spirituality relevant for both religious and non-religious people (Büssing et al., 2018, 2023; Büssing, 2020, 2021). Feelings and phenomena related to awe have been the topic of theologians like Albert Schweitzer and his ethics of Reverence for Life (1965) or Romano Guardini discussing the virtue of awe (1963) but were somewhat neglected by academic psychology. Positive Psychology has been rediscovered in recent years as an object of psychological studies, as a strength of transcendence (Peterson & Seligman, 2004, 537–552), and as a potential for profound transformation (Schneider, 2011). Bucher (2016), in his general report simply puts it as a strength and points out ambivalences if abused by authorities, religious or not. During his career as a researcher, psychologist Keltner spoke to various groups of people about happiness, and he found that all of this “comes down to one thing: finding awe” (Keltner, 2023).

Recent empirical research has found awe perceptions and feelings to be related to the openness towards new experiences in life (Silvia et al., 2015; Yaden et al., 2019; Konaszewski et al., 2022), to mindful awareness (Büssing et al., 2020, 2021), to gratitude (Büssing et al., 2018; Konaszewski et al., 2022), to prosocial behavior and compassion (Rudd et al., 2012; Piff et al., 2015), to engagement for others and the environment (Büssing et al., 2018b; Zhao et al., 2018), to resilience (Konaszewski et al., 2022), to health behavior (Konaszewski et al., 2022), to meaning in life (Zhao et al., 2019, Aschoff, 2021), and to psychological wellbeing (Rudd et al., 2012; Krause & Hayward, 2015, Rankin et al., 2019, Büssing et al., 2021). Awe was further a protective factor against negative perceptions (Koh et al., 2017; Atamba, 2019). During the COVID-19 pandemic, Awe/Gratitude was the best predictor of perceived positive changes in terms of posttraumatic growth, and a resource to recognize the still positive aspects of life (Büssing et al., 2020, 2021).

The experience of awe may be related to the perception of vastness and the need for adaptation and accommodation and thus to low self-focused attention (“smallness”) (Keltner & Haidt, 2003). At least six characteristics of awe perceptions can be defined: changes in time perception, self-diminishment, connectedness, perceived vastness, physical sensations, and need for accommodation (Yaden et al., 2019). A problematic aspect is the perception of vastness and accommodation/adjustment to the experiences, as vastness can be perceived as either great and overwhelming or small and less significant. Depending on the underlying quality of the experience, the related perceptions may require either an adaptation of one´s frame of reference—or no relevant changes in attitudes and behaviors (when it is only a short moment of attraction and aesthetical fascination). The criterion of vastness raised by Keltner & Haidt (2003) might thus refer to rare and exclusive experiences, while most people may nevertheless experience moments of fascination, astonishment, and admiration rather frequently. Using the Awe/Gratitude (GrAw-7) scale in a sample of 7,928 participants, experiences of beauty in general (90%) and experiences of being “captivated by the beauty of nature” (84%) in particular have been perceived often to frequently, followed by feelings of “great gratitude” (77%), becoming very “quiet and devout at certain places” (71%), pausing and thinking of so many things for which one is grateful (71%) while pausing and staying “spellbound at the moment” (60%) and feelings of “wondering awe” (54%) were reported less frequently, yet still by more than half of the respondents (Büssing, 2021).

Feelings of wondering awe are regarded as an emotional reaction toward touching experiences (Keltner & Haidt, 2003; Pearsall, 2007; Silvia et al., 2015; Keltner, 2023). Old interpretations indicate that awe contains elements of fear and wonder (Klein, 2020). The German term “Ehrfurcht” combines the terms of honor and respect (“Ehre”) with fear (“Furcht”). The related emotion might be what Mose experienced in front of the burning bush at Mount Horeb, where he fell on his knees, hiding his face “because he was afraid to look at God” (Ex 3:1–17). However, today, awe is often related to other feelings, such as wonder, and “related states such as admiration, inspiration and elevation” (Stellar et al., 2017). The respective triggers are manifold, ranging from impressive views of nature to charismatic people, childbirth, and the arts (Keltner & Haidt, 2003; Shiota et al., 2007). In former qualitative text analyses where different people stated what caused moments of wondering awe, four main categories were identified: (1) Nature, (2) Persons, (3) Unique Moments, and (4) Aesthetics, Beauty, and Devotion (Büssing, 2021). While some reported perceptions can be regarded as aesthetic fascination, other experiences might have been even more profound. Specific indicators of spirituality were less explicitly stated than expected. One subcategory addressed the perception of the “Sacred in nature” and experiencing “Times of silence”, while another subcategory referred to “Spiritual practices and ceremonies” (Büssing, 2021). One may assume that the specific context of experience and the underlying worldviews, religion, and spirituality influence what attracts someone´s attention, perception, and subsequent interpretation. As awe experiences are related to the frequency of praying/meditation (Büssing, 2020, 2021; Büssing et al., 2023), one may further assume that specific lifestyles and related spiritual practices may sensitize for these experiences and how they can be integrated into the life concept.

For that purpose, we intended to analyze the triggers of awe experiences in a large group of religious brothers and sisters (RBS) with their specific religious lifestyle and regular religious practices in everyday life. It was previously shown that RBS have significantly higher Awe/Gratitude scores than to other groups of people (Büssing, 2020, 2021). In this study, as part of the empirical approach, we first addressed the general experiences of awe and gratitude; in addition, the qualitative part analyses their free text answers in which situations they perceive moments of wondering awe. We expected a wide range of situations that could be interpreted as an awe perception, ranging from moments of fascination and attraction to moments of pausing in wonder, and finally of being deeply moved by the respective experience and subsequent processes of transformation (“adjustment”).

## Materials and Methods

### Participants

### Awe and Gratitude Questionnaire

To address times of pausing in wondering awe in specific situations as a perceptive aspect of spirituality, we used the 7-item Awe/Gratitude scale (GrAw-7) (Büssing et al., 2018). This single-factor scale shows good internal consistency (Cronbach’s alpha = 0.82). Characteristic items are: “In certain places, I become very quiet and devout”, “I stop and am captivated by the beauty of nature”, “I pause and stay spellbound at the moment”, “I stop and then think of so many things for which I'm grateful”. All items were scored on a 4-point scale (0—never; 1—seldom; 2—often; 3—regularly), referred to as a 100% scale.

### Statistical Analyses

Descriptive statistics are presented as frequencies for categorical variables and mean values (± standard deviation, SD) for numerical variables, and subgroup analyses as analyses of variance (Kruskal-Wallis test), using SPSS 28.0. Given the exploratory character of this study, we set a stricter significance level at *p* < 0.01. For analyses of variance, Eta^2^ values < 0.06 are considered small effects, between 0.06 and 0.14 as moderate effects, and > 0.14 as strong effects.

### Qualitative Analyses

Free text field entries were analyzed using qualitative content analysis techniques (Krippendorf, 2004) referring to a phenomenologically inspired approach (Neubauer et al., 2019). To obtain open, experience-oriented narratives, participants were invited to state situations where they experienced moments of wondering awe (“If you have experienced such sensations of pausing in awe, in what situations was it? Please describe this.”). These free-text answers were then coded (deductively) according to the already established categories (Büssing, 2021) and further elaborated where necessary. These codes were combined in a code list and grouped according to their motifs into principal codes (categories) and sub-codes. Representative text passages served as anchor quotations for the respective codes (categories). These German language text passages were translated into the English language.

## Results

### Findings of Quantitative Analyses

Participants (n = 250) were predominantly women (60%) with a mean age of 64.4 ± 14.7 [23–93] years. The largest groups were Jesuits, Franciscans, and Benedictines. All further descriptive data are depicted in Table 1.

**Table 1:** Description of participants (n = 250)

	n	%	Mean ± SD [range]
Gender	250	100	
Women	150	60	
men	50	40	
Age	248		64.4 ± 14.7 [23–93]
Living area	246	100	
Larger city		41.1	
Smaller city		29.7	
Rural		29.3	
Religious Orders/Congregations	249	100	
Franciscans	26	10.4	
Capuchins	11	4.4	
Benedictines	25	10	
Jesuits	44	17.7	
Cistercians	9	3.6	
Carmelites	8	3.2	
Rita Sisters	9	3.6	
Salesians	6	2.4	
Sisters of Mercy	9	3.6	
other	102	41	
Contemplative or active Congregation	249	100	
Predominantly Contemplative	51	20.5	
Predominantly active	198	79.5	
Awe/Gratitude (GrAw-7) mean score	249		70.2 ± 14.8 [0–100]
ED1 I have a feeling of great gratitude			2.2 ± 0.6 [0–3]
ED2 I have a feeling of wondering awe			1.9 ± 0.7 [0–3]
ED3 I still have learned to experience and value beauty			2.4 ± 0.5 [0–3]
ED4 I stop and am captivated by the beauty of nature			2.2 ± 0.7 [0–3]
ED5 I pause and stay spellbound in the moment			1.8 ± 0.7 [0–3]
ED6 In certain environments/places I become very quiet and devout			2.1 ± 0.7 [0–3]
ED7 I stop and then think of so many things for which I'm really grateful			2.1 ± 0.7 [0–3]

The Awe/Gratitude mean score is 70.2 ± 14.8 [0–100], thus located in the upper range (Table 1). Pausing “spellbound at the moment” and “feeling of wondering awe” scores are the lowest (albeit high anyway), while the experience and value of beauty, in general, scored highest (Table 1).

Awe /Gratitude was reported significantly more frequently by women than men, while there were no significant differences for age categories or between RBS who are predominantly contemplative or predominantly active religious (Table 2).

**Table 2:** Frequency of Awe/Gratitude perceptions in subgroups

	Awe/Gratitude (GrAw-7)	
	Mean	SD
All participants	70.17	14.75
*Gender*		
Female	73.53	14.74
Male	65.09	13.30
Kruskal-Wallis H value	19.65	
Eta^2^ value	0.079	
*p* value	< 0.001	
*Age categories*		
< 51 years	65.32	14.80
51–60 years	69.59	15.35
61–70 years	70.50	14.87
71–80 years	73.13	16.63
> 80 years	72.74	11.42
Kruskal-Wallis H value	8.05	
Eta^2^ value	0.032	
*p* value	0.090	
*Contemplative or active congregation*		
Predominantly contemplative	69.69	14.47
Predominantly active	72.76	14.94
Kruskal-Wallis H value	1.72	
Eta^2^ value	0.007	
*p* value	0.190	

### Findings of Qualitative Analyses

Triggers of these experiences were reported as free text statements by 81% of participants. These triggers of awe were related to six main categories: (1) Nature, (2) Special Moments, (3) Transcendence Perceptions, (4) Religious practices, (5) Distinct People, and (6) Aesthetics, Art, and Culture. In the following, the main categories and their sub-categories will be described, and representative text passages that serve as anchor quotations are added (with anonymized IDs of the person, gender [f for females and m for males], and age).

Apart from the content analyses, the number of statements was also counted. Awe triggers related to Distinct People (184), Nature (161), Religious practices (126), and Special Moments (124) were most often mentioned, less often Aesthetics, Art and Culture (74) and least Transcendence Perceptions (35).

### Nature

#### Experience of Nature

Most participants stated “nature “ or “beauty of nature” as triggers of awe. Specifically, green and blue spaces were stated (“hills”, “ocean”, “lakes”, “landscapes”), but also specific animals and flowers, sunset, rainbows, and the play of light. Specific statements are: “in front of a beautiful landscape; at a sunset “ (m39), "in nature like now in the autumn forest when the light falls through the leaves" (m48), "Primarily in nature—on mountain tops—in gardening—at the appearance of a rainbow “ (m55), "through blooming landscapes, by a river, by the sea, at sunrise or sunset “ (m75), "when I stand at the open window in the morning and hear the birds and the sun comes through “ (f70). One may assume that these experiences were foremost a matter of fascination.

Indeed, moments of fascination were mentioned in three statements, “Today, I observed a deer in a harvested corn field, and it played with the sun, alternating between shadow and light. That fascinated me for a moment.” (m63). A religious sister stated, “When the sun rises and shines through the windows; when I look at the starry sky at night; when I see a fascinating animal that I have never seen before.“ (f37). It was also perceived that this beauty of nature is threatened, “While staying in nature, I am fascinated by its beauty, although I now also feel their groaning; forests from my childhood days are completely destroyed by bark beetles and environmental influences.” (f71).

Some of these experiences are triggered by the vastness of the sky or the infinity of the universe in particular: "I lie with my back on the water and look up at the sky." (m61), “When I go for a walk at night and look at the starry sky.” (m68), "… the vastness of space, the universe, millions of Milky Ways “ (m68).

#### Perceiving the Beauty and the Sacred in Creation

Being attracted by the beauty of God´s creation and resulting feelings of happiness are stated clearly: “The beauty of creation, sea, mountains, flowers in their diversity, always amazes me and makes me happy about it from the bottom of my heart.” (f85). Some participants relate these experiences to the Sacred or God as creator: “When I see the details of creation, it is unthinkable that there is no God, no human being can make that so wonderfully incomprehensible.” (f35), or, “God's creation is so wonderful. I can only marvel.“ (f78).

These experiences may elicit a deep longing that cannot be satisfied: "Deep experience of the beauty of nature—at the same time the feeling that this experience cannot still the longing it arouses in us.“ (f65).

### Special Moments

#### Moments of Quietness

Moments of quietness and silence were mentioned as awe triggers: “during times of silence” (m56), “being alone in silence” (m55), “while staying still” (f84), “enjoying silence” (m55). Such special times of quietness and silence may be more easily perceived in specific places, either in nature or in quiet church rooms, for example: "The silence in a forest.“ (m85), „Silence and solitude in wooded areas.“ (f79), “The silence in small chapels and Romanesque churches.“ (m64), „In a church room that breathes silence.“ (f61). One woman is attracted and emotionally touched by the opposite of vastness, "Again and again churches, especially their interiors with charisma, small inconspicuous and quiet places “ (f65).

These moments are not necessarily experienced alone, but also in the religious community: “Becoming quiet in the community” (m80) or, “At a silent day in the community” (f80). Inner quietness is often stated as the cause of awe experience, particularly during intentional periods of rest, „It comes mostly when I take a little break and pause.“ (m46) or, „When I come to rest (e.g., during meditation, in retreats, when listening to music and singing).” (f55).

These moments of touching quietness can be further experienced during prayer times (f50, f68, f72) or “In silent worship “ (m58, f56). Spiritual practices as awe triggers will be further elucidated later on.

#### Moments During Everyday Life

Awe was not only perceived during quiet times and holidays (f79, m56, m56, f65, f70) but also during everyday situations that suddenly stand out as special: „In the very banal and normal everyday life.“ (f70), "The little things along the way.“ (m64), „In everyday life with a cup of coffee.” (f59), „Under a hot shower, after a restful sleep, a cup of coffee in the morning.“ (f52). Such statements of mindful perceptions may widen the view for a “greater picture” (which will be addressed later on): “The small and easily overlooked often gives me an idea of the actual beauty and connectedness.” (f54).

#### Reflection of Life and Related Insights

Such moments may facilitate reflective processes. Looking back in terms of a reflection on the day helps to recognize specific moments regarded as moments of awe (m53, m39, m48, m56, f66, f72). This means that participants recall what has attracted their attention and should be memorized as special, “Often in retrospect. I keep a journal to record such experiences of being guided.“ (f60).

The perceived meaningful interconnectedness of situations and encounters was stated as awe triggering, with subsequent specific insights that amplify awe,”If difficult personal experiences for which there was only a "Why?" made suddenly sense, because they make it possible to understand a person in a similar situation and to be of assistance to them.“ (f73), “In retrospect. How things come together. When processes suddenly seem reasonable. (…) When you suddenly have the feeling that it's good enough the way it is.“ (m32), ”Suddenly I know how everything is connected.“ (f65). These insights may also confirm the conviction that God cares even in difficult life situations: „Looking back at difficult situations when I realize that God and people were there, after all.“ (f35), or, “The insight that one is exactly as God wanted me to be: When I realize that I am "a premiere on the stage of life", that I am absolutely wanted by HIM who created me.“ (f81).

One sister stated that most situations, experiences, and encounters fall into place like unexpected gifts,”Also looking at my own life and all the events that happened, the wonderful people I was able to win as friends, with all the gifts that fell into my lap unplanned and deliciously.“ (f73). In another statement, it is the perception of life as a miracle:”Sometimes the wonder of just being there overwhelms me… “ (f81). Both statements are not direct perceptions of pausing in wondering awe, but reflective insights in retrospect. Thus, this reflective aspect could be regarded as a second-order experience in terms of *wondering* rather than a first-order experience of awe (Gallagher et al., 2015).

#### Coping with Difficult Situations

As mentioned before, the conviction that God is with them both in the everyday challenges of daily life duties (m23, m46, f70) and also in difficult life situations (f68, f93), is regarded as awe-inspiring in retrospect. Moments at work when things went well, or something was achieved were stated, “Sometimes especially in the moments when I am very tired and exhausted from my tasks. But these have fulfilled me very much.“ (m46), „After work done; when something is achieved.“ (m23), “After difficult tasks which, with God's help, have been successfully completed.“ (f53).

This motive of overcoming was stated in some further quotes: „Personal situations of success, relief, relaxation after a big challenge.“ (f65), “When something difficult is done.” (f66), and „When tensions resolve in unexpected ways.“ (f55). The motive of overcoming was also referred to difficult times of illness: „In some situations when ‘new life’ was given to me after a very difficult time.“ (f68), or, „After surviving illness.“ (f93).

#### Gratitude

Gratitude is often the consequence of awe perceptions, amazement, and reverence. They may be perceived simultaneously: „For this reason, there is no pure gratitude and wonder.“ (f49), "Amazement and gratitude for the greatness and love of God that accompanies my life.“ (f75), "Gratitude and reverence in the memory of my parents, for their guidance, their accompaniment.“ (f80).

New insights may widen the horizon, resulting in gratitude, wonder, and reverence: „I'm amazed when an unexpected experience, a look at nature, a new knowledge broadens my previous horizons. At the same time, my horizons proved to be too narrow, too one-sided, too naive. For this reason, there is no pure gratitude and wonder. Reverence is also sobering because I experience that I'm not the best, the smartest, the greatest.“ (f49).

In other cases, the motive of gratitude is at the forefront. These statements will not be further explored, i.e., “Acting on the day's reflection in gratitude for relative health and the opportunity to work. Gratitude for "coincidences" about how things come together”. (m55), “When I think about my long life of 86 years and many experiences, I can only marvel and am very grateful. I have been in this order for more than 60 years, and despite all the ups and downs, God has kept me in his relationship with him. I can only thank God for that.“ (f86).

### Transcendence Perceptions

A complex and heterogeneous pattern of awe triggers refers to the field of perceiving the Sacred in different life concerns. This requires a reflection of life (2.3), where God as a ‘Director’ is in the foreground.

#### Presence, Guidance, and Work of God

The perception of being guided and sheltered by God raised awe perceptions in retrospect: "When I experience myself in my everyday life, how God takes care of me by showing me the direction through an inspiration, sending me unexpected help and the like.“ (f55), "Review of my whole life. In my depressions, pain, weaknesses, disappointments, lack of understanding, providences, I recognize more and more God's help and loving companionship.“ (m85), "In thinking about my life and the single stations; I feel the guidance of God and my trust is strengthened.“ (f71), "In experiences of providence and guidance (being touched by "heaven")." (f65).

In some cases, it was the reflection of the life of religious ancestors where the guidance of the Holy Spirit was perceived, that was indirectly or directly expected for their own life, too: “Work of the Holy Spirit in the history of the order and church.” (m38), “When I become aware of the work of the Spirit in life stories.“ (m80), “Action of God in the lives of many people, especially the saints and martyrs. How God carries and guides me.“ (m56).

Other perceptions were that God is responding (“When God answers “, f41), or is still present in the different courses of life: “When I become aware of the presence of God, He is the I-am-there; when I receive unexpected "gifts" from God, especially in times of depression, e.g., a beautiful sunrise, gentle rain or wind." (f68), “Review of my whole life. In my depressions, pain, weaknesses, disappointments, lack of understanding, providences, I recognize more and more God's help and loving companionship.“ (m85). “Often I am amazed, grateful and fulfilled when I see how this "unwaveringly faithful God" guides and accompanies us.“ (f72).

In other cases, mostly religious sisters reported that situations resolved themselves and suddenly everything fell into place (f49, f65, f65, f66, f68, f68, f71; f73, f76, f84, f88, m85): "When, after heartfelt prayer, problems are suddenly resolved with joy.“ (f68), or, "How some seemingly hopeless situations can still change for the better.“ (f76).

One statement explicitly points to a comforting (divine) light: "Light; I often experience it as "brightness of the soul", and then I know that consolation carries me.“ (f49).

#### Connectedness/Vastness

This topic is quite diverse in its underlying perceptions. It includes wideness, beauty, meaningfulness, interconnectedness, and unity: “The small and easily overlooked often gives me an idea of the actual beauty and connectedness. Sitting by a babbling fountain that pulls you into oneness.“ (f54), “I admire the perfection, the beauty, the harmony—even as it fades away. Meaningfulness and security seem obvious there, without a doubt. Again and again, I experience this unity when pausing at my desk, or on the way to work “. (f60), “When I feel deeply connected to people—to God.” (f68).

The motive of breathing was explicitly stated in some quotes which indicate feelings of vastness, “When my breath and the breath of the forest become one.“ (f75), “The space surrounds me, my breath perceives it, the depth and height, various dark and light areas. I feel like I can take in the space with my breath.“ (f81).

As a result, new insights about the own perspectives may arise: “I'm amazed where an unexpected experience, a look at nature, a new knowledge broaden my previous horizons. At the same time, my horizons proved to be too narrow, too one-sided, and too naive. For this reason, it is not pure gratitude and wonder. Reverence is also sobering because I experience that I'm not the best, the smartest, the greatest.“ (f49).

### Religious Practices

Religious practices are an essential part of the daily life of RBS. Therefore, various statements mention prayer and meditation as triggers of awe experiences, while other practices and triggers were mentioned, too.

#### Prayer/Meditation

The different forms of prayer were stated very often, specifically the morning prayer (m71, m81) and evening prayer (m23, f66), and the Liturgy of Hours in general (m38, m57, m74, f55, f65, f83, f84); prayer in silence (f50, f68, f72), contemplative prayer (f59, f68, f73), the Our Father (m86). In addition, times of (silent) meditation were often mentioned (m38, m54, m57, m86, f56, f65, f72, f73, f93). All these were associated with feelings of awe.

Religious music and songs as a specific form of prayer were mentioned as triggers of awe, too: "With beautiful music at church services.“ (m48), "In certain chants, e.g. hagios theos.” (m71), "When singing, especially religious songs, including Gregorian chant.“ (f63), "While singing the Psalms.” (f66).

#### Holy Sacrament

Worship of the Holy Sacrament as a specific form of contemplative prayer was stated explicitly (m44, m54, m58, m63, m80, f56, f63, f68, f83). One sister underlined that the insights related to times in front of the Eucharist raised feelings of awe: "Especially in adoration: what a great gift the Holy Eucharist is: in such an inconspicuous sign the Lord of all the world and time comes to us, who bears and preserves everything.“ (f55).

The celebration of the Eucharist is stated as a trigger of awe (m50, m70, m74, m80, f55, f63, f80) as well as liturgy in general (m23, m38, m41, m86, m57, m57, m80, m86, f49, f56, f57, f62, f65, f66, f70, f71, f80, f82, f83, f84, f93). However, no specific information was given about what exactly the touching experience was.

#### Scripture Reading

Reading and contemplating the Holy Scriptures may sensitize to moments of awe (m74, m75, m90, f46, f56, f65, f75, f80). Here, the words may resonate with the own life: “When a word speaks to me, e.g. from the Holy Scriptures.” (f46), “Always amazed at how texts from the Bible affect my life.“ (f75), “Sometimes, when an inner light dawns on me in Scripture or other books, I can feel personally addressed.” (f66).

#### Retreats and Pilgrimage

Retreats with their specific religious input were stated as triggers of awe (m39, m47, f65, f66, f73, f74, f85), which implies quiet times of reflection (f55, f77). These reflections help to reconnect with God: “Also in retreats, I am impressed to experience that God cares about me. I'm the center of his attention.” (f60), "When God inspires me for my work; when I experience, e.g. in a retreat, how God cares about me and leads me.“ (f61), “At retreats—we, I am loved by HIM.“ (f80).

Reflexive times of pilgrimage were mentioned as awe triggers (m47, m48, m65, m84), specifically the Way of St. James (m48, m74, m84).

### Distinct People

During encounters with distinct people (historical or temporary), one can be attracted by their charm, impressed by their actions and sayings, or fascinated by their skills (i.e., as artists). Whether being impressed is a matter of wondering awe or rather some kind of attraction, is often unclear. In other cases, these distinct people are not extraordinary, as it was the simple presence of close friends and family or children, in general, that was remembered, or their encounter with suffering or dying people that impressed the RBS.

#### Intensive Encounters

Intensive encounters and talks with others in general, either friends and family or within the religious community, were mentioned particularly by religious sisters (m41, f38, f49, f55, f58, f61, f64, f70, f73). They often used the term “connection” to underline that something happened between two people that they did not expect in that way. Two statements underline that these encounters can also happen with “unexpected guests” or “strangers” when they felt connected with them during their talks, “Encounters with people, often strangers, when a connection happens through a gaze, a smile.“ (f58), or, “When unexpected guests come and a conversation is endlessly beautiful.” (f64).

Meeting with people at the margins of society can also trigger awe by perceiving an unexpected connection: “I meet a homeless person, and we start talking and are simply connected as equals.” (m59), “In encounters with the poor and sick, people at the margins of society.” (f29), “In dealing with people (especially those who are much worse off than me/us). (f55).

Sometimes, people show a new side that impresses them: “When people are completely different than I thought.“ (f65), “Encountering a fellow human being whom I suddenly saw in a completely new light.” (f65).

#### Extraordinary People

Considering the life of extraordinary people and saints led to astonished admiration in some RBS: “Extraordinary biographies in the history of the order and the church.“ (m38), “The witness of faith in people who lived before and with me.” (m64), “I have often been able to experience the fraternity in Taizé (Burgundy). I could also meet the founder, Frère Roger.” (f84).

However, also the extraordinary behaviors of ordinary people impressed RBS, “When people who have little money share generously anyway!” (m44), “In conversations with people who have often had the hardest fates and yet do not despair, but instead work for what is important to them.” (w81). Participants were also impressed by people who stand their ground against other opinions, “When people commit themselves selflessly; when people follow their inner trail—regardless of the praise/negative criticism of others.“ (f66), “I experience a young politician and am frozen in awe at how committed and loving she is to the problems of her city.” (m59), “When people get involved in church today—for more freedom, synodality, for the LIFE message of God.” (f66).

Moments of fascination because of specific skills were mentioned, too: “When I see people doing "tricks" (acrobatics, painting, sewing….).” (f58).

#### Impressive interactions

The way people behave and interact are stated as awe triggers. Here one may assume that the RBS were foremost impressed, and interpret it as awe only in retrospect: “When people meet lovingly.“ (f52), “Observing people who treat each other lovingly.” (f61), “When I watch old people and see them happy, as well as young couples…. and much more…” (f58), “When I hear that good things have happened to others—when I experience how people support each other—when people forgive each other and peace reigns again.” (f70).

Overcoming conflicts are causes of wondering, too, “I experience a conflict and can be there when everything is said and both parties find a new solution.” (m59). Processes of reconciliation can be seen in the same context: „When forgiveness and reconciliation occur.“ (f55), “In mutual understanding, in signs of closeness and affection, in the experience of forgiveness and reconciliation, in perceiving everything that has been given to me.” (f65). The motif of reconciliation was also used in the context of life review, which could be regarded as an encouraging inspiration for one´s own life: “When old people look back on their lives with reconciliation; if reconciliation is possible—on a small or large scale.” (f66).

#### Birth/Children

Children and how they react and interact, their spontaneity, and unconditional trust (m64) impressed (mostly female) RBS. Children in general were stated (m57, m80, f49, f51, f52, f55, f65, f68, f73, f74, f80), particularly playing children (m71, f58, f60, f66).

Further, the birth of a child (f59), watching (f61, f71, f84) and holding a newborn child (f45, w65, w87) was stated as awe-inspiring. For one religious sister working as a midwife, each birth is something extraordinary beyond ‘control’, “As a midwife, I am fortunate enough to marvel at the many newborns. Birth and the beginning of life remains a fascinating event. I can only marvel and thank.” (f65).

Similarly, religious sisters who have no own children yet care for children experience moments of awe: “I work as a doctor in Africa, and looking deep into children's eyes is a moving experience for me, which makes me happy, amazed, and gives me strength. Most small children are still very open, and in these moments, an intimate, albeit brief, connection is formed.” (f65), or, “I live in the children's home. I often meet a happy child. His trusting eyes amaze me.” (f86). In these statements, the “intimate” connection with the children in their care is clearly expressed.

#### Sickness, Suffering, and Death

The other polarity to birth and new beginnings is illness and death. Confronted with sick, suffering, and dying people, participants are touched and moved by moments of grief and farewell. Some of these experiences were made in the context of their pastoral work and spiritual accompaniment (m77, m81, f46, f55, f65, f66), “In the accompaniment of the dying, in the spiritual accompaniment, especially of grieving young people who have experienced very difficult things and often deal with their wounds so courageously!” (f65), "Special awe as someone literally breathed their last in the ICU when suddenly the screen went black and there was a sacred silence in the room.” (f66).

In other cases, participants report that they are impressed by the strength of the suffering and dying, “With what trust in God people accept their suffering and their difficulties.“ (m64), “How much strength people have to endure suffering without despair.“ (f76), “I am with a very elderly person and I see his zest for life in the face of death.” (m59), “Yesterday at the bedside of an AIDS/HIV patient (…): he knows that he will die soon and absorbed the words of Jesus with wide-awake eyes.” (m61).

A longer statement underlines the complexity of the experience: “Just now, when a fellow sister, terminally ill, was able to look back on her life with such great gratitude, and even though she had experienced so incredibly difficult things during the war (escape, two siblings died in her arms, separated from their mother, at the age of 14 she took responsibility for her siblings etc.), not a bit of bitterness but a happy heart that was ready to give her life back into the hands of God and died with a smile on her face.” (f56).

### Aesthetics, Art, and Culture

Music, art, and architecture attract people in different ways. It can be an esthetical fascination, admiration of the skills of a particular artist, but also a profoundly moving experience where the ‘work’ resonates with the perceiver. In the following paragraphs, such triggers are described.

#### Art, Music, and Literature

Moments of wondering awe were perceived in museums while viewing the work of certain artists (m45, m49, m51, m75, f58, f60, f65, f74, f80, f84), while going to concerts or listening to music in other ways (m45, m57, f49, f55, f58, f59, f60, f70, f71, f74, f91), when someone is singing (m58, f54), while reading books (i.e., poetry, bible, religious/theological books) (m74, m75, m86, f49, f65, f70), watching movies (f56) or going to theater (f58). Here, a resonating attraction seems to be relevant, “While listening to a wonderful melody.“ (f59), “With wonderful music, when the fullness of the music covers the whole body (e.g. organ).” (f64), or “With music, which often hits the heart.” (f85).

#### Sacred Buildings/Places

Churches and other sacred buildings were often mentioned as causes of awe perceptions (m50, m51, m51, m60, m63, m71, m73, m80, f37, f48, f52, f60, f62, f66, f71, f74, f84, f87). The inner “charisma” of these buildings attracts the RBS; it evokes specific feelings, “Again and again churches, especially their interiors with charisma.” (f65) or, “In some rooms that radiate the harmony and presence of something bigger.” (f68).

There are also more specific places related to distinct charismatic people:”When remembering great personalities, especially in places associated with them (e.g. the sites of the assassination on Bishop Oscar Romero or of the execution of Alfred Delp (Ploetzensee) and Dietrich Bonhoeffer (Flossenbürg).” (m74).

Also, architecture itself attracted attention, related to both sacred and profane buildings, “In front of a monumental building (cathedral, high-rise building, monument, etc.).” (m75),”Architecture (Romanesque, Gothic or very austere modern churches).“ (m52), “looking at certain buildings: churches, lighthouses” (f66). What exactly attracted them, remains unclear; maybe it is simply a fascination of their beauty, as it was stated by two religious sisters in general („at the sight of beauty “, f52; “if I am allowed to see beautiful things (nature, people, liturgy, art)” (f55).

#### Own Creativity

Finally, the process of being creative oneself was stated as an awe-triggering experience: “I write a text and am amazed for another 20 min how it turns out.” (m59), “Being creative yourself.” (f58), “When making music, especially on the organ.” (f93).

## Discussion

The RBS enrolled in this study scored high on Awe/Gratitude, which was nevertheless 6 points lower (70.2 ± 14.8) as compared to another sample of RBS and priests (76.1 ± 16.1) recruited before the COVID-19 pandemic (Büssing, 2020, 2021). Also, in this group, moments of pausing and being attracted by something were reported more frequently than specific moments of wondering awe, and being “spellbound at the moment”. This means there are differences in the frequency of the respective perceptions that all may relate to feelings of wondering awe and admiration, rather than fear. The motive of fear was not explicitly found. However, not all of these perceptions might be ‘pure’ awe perceptions.

In this study, we asked RBS to recall perceived causes of awe perceptions in their life, without defining what awe exactly is and how it should be interpreted. Therefore, their perceptions and interpretations are relevant. As a consequence, we have a spectrum of situations that were interpreted as moments of awe, ranging from attraction (attention or fascination) and moments of pausing in wonder (astonishment) to being deeply moved by the respective experience (awe). These can be either short-term experiences that interrupt the flow of routine without needing adjustment, or they are reflective processes in retrospect that result in new insights, attitudes, and behaviors (indicating spiritual transformation). Therefore, Gallagher et al. (2015) differentiated reflective aspects as second-order experiences of *wondering* from immediate first-order experiences of *awe*. Bucher (2016) recalls the closeness and differences of awe-related phenomena as put by the German philosopher Otto Friedrich Bollnow (1903–1991): fear, respect, esteem, admiration, and amazement.

### Description of Awe Trigger Categories

Compared to the topics and categories found in a more general sample (Büssing, 2021), the responses of RBS with their specific religiosity have changed the context of categorized awe triggers. While Bucher (2016) used 62 predefined “objects” of awe that were categorized according to factor analysis, resulting in eight categories (Table 3), we referred to spontaneous answers of people what have triggered their awe perceptions without defining them. After content analyses of these open responses, the four main categories identified by Büssing (2021) were extended to six main categories according to the data in this sample of RBS. In Bucher´s study, the strongest triggers were reverence for life in general, people with civil courage, a concentration camp memorial, a high mountain peak, and a newborn child (Bucher, 2016). These perceptions may be interpreted as amazement, respect, shock, and admiration. In our current study, with its six categories, the related sub-categories became more differentiated (Table 4). *Transcendence Perception* is one of the new categories, while the other new category *Religious practices* was part of the former subcategory “Spiritual practices/Ceremonies” related to *Aesthetics, Art, and Culture *(Table 4). Both topics refer to the specific lifestyle and related spiritual practices of RBS. Within the main category, *Special Moments*, the sub-categories were slightly adjusted and two new ones were added (e.g. Coping with Difficult Situations and Gratitude). All other sub-categories can be considered as similar in principle, with some thematic adjustments.

**Table 3:** Related main (and sub-) categories of awe triggers as analyzed in Bucher’s (2016) sample compared to the categories in Büssing (2021) and found in this study with RBS

Bucher (2016) (factor analysis)	Büssing (2021) (content analysis	This study with RBS (content analysis)
Threatening natural forces	–	–
Majestic beauty of nature	Nature	Nature
Life, socially close persons	Distinct people	Distinct people
Morally outstanding, courageous persons	Distinct people	Distinct people
Dead people and their last resting places	–	–
Religious and church-related aspects	–	Religious Practices
National symbols	–	–
Fame, Power, Richness (wealth)	Distinct people	Distinct people (Extraordinary people)
–	Aesthetics, Art, and Culture	Aesthetics, Art, and Culture
–	Special moments	Special moments
–	–	Transcendence perceptions

**Table 4:** Identified main categories and related subcategories of awe triggers

Main Categories	Sub-categories in general Büssing (2021)	Sub-categories in RBS
1. Nature	•Experience of nature	•Experience of nature
	•Perceiving the sacred in nature	•Perceiving the beauty and the sacred in creation
2. Moments	•Uniqueness of the moment	•Moments of quietness
	•Times of silence	•Moments during everyday life
	•Moments of understanding	•Reflection on life and related Insights
		•Coping with difficult situations
		•Gratitude
3. Perceptions		•Presence, guidance, and work of God
		•The Big Picture/Connectedness
4. Practices		•Prayer/Meditation
		•Holy Sacrament
		•Scripture reading
		•Retreats and Pilgrimage
5. Distinct People	•Encounter with outstanding, inspiring examples	•Intensive encounters
	•Birth/Own Children	•Extraordinary people
	•Accompanying the Dying	•Impressive interactions
	•Miracle of Man´s Organism	•Birth/Children
	•Professional Situations: Crises	•Sickness, Suffering, and Death
6. Aesthetics, Art, and Culture	•Aesthetics/Beauty	•Art, Music and Literature
	•Sacred Buildings	•Sacred buildings/places
	•Spiritual Practices/Ceremonies	•Creativity

Some of these sub-categories point to immediate experiences of attraction and awe, i.e., the Experience of nature, the Perception of its beauty, and the Sacred in creation. Also, moments of quietness and silence may facilitate the immediate perception of the Sacred. These special moments can be perceived during the quiet times of prayer and meditation, and while contemplating the Sacrament. Such moments of quietness do not mean the absence of sound, words, or other people. Instead, moments of inner quietness and reflection during shared times of religious practice are meant (i.e., recitation of psalms, worship of the Sacrament, singing religious songs within the religious community). In addition, moments of emotional connectedness with other people and everything around (“nature”) were perceived as moments of awe. RBSs´ feelings of connectedness could indicate some kind of transcendence conviction that God is present in everything and could thus be experienced in all moments. Feelings of unity and vastness can be the consequence. The experience of connectedness is a core dimension of the multidimensional construct of spirituality (e.g. Engebretson, 2004; Puchalski et al., 2014).

Other categories refer to insights and perceptions in retrospect, when special moments were reconsidered and seen in a broader context (i.e., Reflection on life, Coping with difficult situations, Encounters with suffering and dying people). The result of these reflections is wonder, which is congruent with the second-order experience of Gallagher et al. (2015). The admiration of extraordinary people of the past could be a matter of being impressed and thus admiration of these people in retrospect; their behavior may encourage RBS to follow their examples. Also, the immediate encounter with ordinary (everyday) people can trigger feelings of astonishment because of their behaviors (i.e., Impressive interactions), or their strengths and virtues (i.e., Encounters with suffering and dying people). Again, participants wonder and may reflect on whether they would behave similarly in such situations. The birth of a child (“holding a newborn”) was perceived as an immediate moment of wonder, fascination, and awe as such a moment marks the “beginning of life” where one “can only marvel and thank”, as stated by a 65-year old religious sister working as a midwife. During that time, a ‘sacred moment’ interrupts the profane routine, and the only reaction is to wonder (“I can only marvel and thank”). Also, in qualitative interviews with women who gave birth to their child, a “sense of awe at the creation of a new life within the context of birth” was stated (Callister et al., 2001).

The category *Aesthetics, Art, and Culture* addresses moments of fascination and admiration related to the outcomes of various creative processes (i.e., Arts, Music, Literature, Sacred buildings, and architecture) that require skilled people as creators. These outcomes can attract and impress people, and one may stand in astonishment and admiration when one is immediately touched.

### Awe as an Experience of the Sacred Other and Self-Transcendence

The frequency of stated awe triggers shows a wide variety. Most often mentioned were *Distinct People* (184) and *Nature* (161), further *Religious practices* (126), and *Special Moments* (124), while *Aesthetics, Art, and Culture* (74), and particularly *Transcendence Perceptions* (35) were less often stated. The perceptions of the category *Transcendence perception* include RBSs´ feelings of being guided by God, of God´s presence and their perception of being guided, and finally, of being connected with everything around (referring to the awe criterion of vastness stated by Keltner & Haidt, 2003). These are extraordinary perceptions in terms of religious ‘peak experiences’. Generally, peak experiences (Maslow, 1964) may occur during different situations, i.e., religious, nature experiences, intimate moments with friends or family members (Polyson, 1985), as they were described as awe triggers herein, too. Maslow regarded these peak experiences as essential for self-actualization, which means developing all intended abilities and appreciation for life, resulting in new views of the world and oneself (Maslow, 1962, 1964). Self-actualization is a lifelong development process that requires reflection and reinterpretation of one’s own experiences (Rogers, 1951). Positivistic as this concept of self-actualization may seem, it is useful for interpreting the secondary perceptions of wonder, resulting in new insights, attitudes, and behaviors. Interestingly, self-actualized individuals have a tendency (in theory) to be more grateful and more sensitive towards extraordinary moments, and they sincerely appreciate the blessings in life (Maslow, 1954). This is what the outcomes of awe experiences usually are.

As awe and gratitude are regarded as self-transcendent emotions (Stellar et al., 2017), ego-centered behaviors may become less dominant as compared to prosocial behaviors. Awe was found to be related to prosocial behavior and compassion (Rudd et al., 2012; Piff et al., 2015), and engagement with others and the environment (Büssing et al., 2018b; Zhao et al., 2018). Of course, such a relationship does not exclude cases and feelings of awe as part of the dynamics in narcissistic feelings of grandiosity.

Self-transcendence in the authentic sense of the word, however, implies an awareness of interconnectedness, and in most cases, secure attachment and self-actualization (Reischer et al., 2021). The interpretation of self-transcendence as being an integral part of everything around (“cosmos”) relates to the sub-category of Connectedness/Vastness of the main category of *Transcendence Perception*. In contrast to non-religious people, RBSs refer to these perceptions as the presence of God, who has created everything and can be experienced in everything around.

#### Awe and Wellbeing

Awe perceptions were found to be related to subjective well-being (Rudd et al., 2012; Krause & Hayward, 2015; Rankin et al., 2019; Büssing et al., 2021), and several of the identified triggers herein are by themselves related to psychological wellbeing. While subjective wellbeing may facilitate openness towards awe perceptions, awe perceptions may by themselves increase satisfaction with life and thus wellbeing. During the pandemic, the resource Nature/Silence was the mediator of the link between Awe/Gratitude and psychological well-being (Büssing et al., 2022). Enjoying moments of silence and contemplation was associated with personal growth, relaxation, well-being, and psychological stability (Goal et al., 2014; Sampaio et al., 2017; Naor & Mayseless, 2020). Further, people who are more aware of nature as a resource and feel connected with the natural environment have stronger feelings of happiness and well-being (Mayer et al., 2009; Nisbeth et al., 2011; Capaldi et al., 2014). Nevertheless, nature connectedness differs among people (Mayer & Frantz, 2004), and this may also be true for being aware of nature´s beauty and being attracted by it. Awe perceptions vary significantly between different groups. With varying effect sizes, older people (Cohen´s d = 0.80), women (Cohen´s d = 0.32), and people with a religious background (Cohen´s d = 0.18) score significantly higher on Awe/Gratitude as compared to younger people, men, or people without a religious background (Büssing, 2021). One may assume both, differences in (a) life experience and changing priorities and (b) religious socialization and spiritual awareness. Individuals who perceive moments of awe and gratitude may behave differently in comparison to individuals who do not perceive it or only to some limited extent.

It is important to underline that the variety of awe triggers is quite large. Different people are thus attracted by different situations. Despite such differences in general and differences in the intensity of attraction in specific, people nevertheless seem to prefer specific characteristics of nature, particularly trees, shrubs, and water, and some kind of unexpectedness (‘mystery’) within an impressive landscape (Kaplan, 1992; Kuo et al., 1998; Astell-Burt & Feng, 2021). Awe perceptions are related to psychological well-being (Rudd et al., 2012; Krause & Hayward, 2015; Rankin et al., 2019; Büssing et al., 2021) and prosocial and pro-environmental behaviors (Rudd et al., 2012; Piff et al., 2015; Büssing et al., 2018b; Zhao et al., 2018). Awe/Gratitude perceptions were relevant predictors of positive changes in attitudes and behaviors during the COVID-19 pandemic (Büssing et al., 2020, 2021). Consequently, one could intend to train the ability to be aware of special moments in life (and thereby refer to it as a resource). During the pandemic, awe was particularly related to the factors of Nature/Silence/Contemplation, Spirituality, and Relationships (Büssing et al., 2021), which indicates “higher awareness of a connectedness with the world around and with concrete others (horizontal direction of relations) and with the Sacred (vertical direction of relations)” (Büssing et al., 2021). The perception of nature connectedness with its positive impact on well-being and prosocial behavior was tested in environmental training programs (Pirchio et al., 2022; Down et al., 2022). Training in mindful awareness was found to have positive effects on mental health indicators (Janssen et al., 2018; Kriakous et al., 2021) and further improves appreciation of beauty, love, gratitude, and spirituality (Pang and Ruch, 2019).

### Limitations

RBSs were invited by email to participate in an online survey, and not all may have been reached by this route of information. One could suggest that particularly the elderly religious, who are assumed to be less familiar with the use of the internet, were not reached; bearing this in mind, it is remarkable that at least 21% of our participants were 80 years of age and older, indicating that several of them are using internet resources.

We cannot exclude the possibility that only those RSB have participated who regard their awe experiences as relevant enough to be reported. At the same time, those who may regard their experiences as ‘too small’ might not have responded.

When RBS regarded their experiences as moments of awe, we will accept this. We have not asked whether these experiences have significantly changed their lives, and we did not differentiate moments of aesthetical fascination and wondering awe, which might be qualitatively different. Nevertheless, both could be related to subsequent changes in awareness and well-being. This should be addressed in future in-depth qualitative studies.

Of course, people from other cultural or religious societies may perceive moments of wondering awe differently and their triggers may differ from those reported here.

## Conclusions

Because of their religious lifestyle and related spiritual practices, related to awe perceptions, RBSs´ statements about what triggers their awe perceptions have extended the previously reported categories of awe triggers (Büssing, 2021). Particularly *Transcendence perceptions* and *Religious practices* are new categories. Although it is difficult to assess whether their perceptions are moments of aesthetical fascination or other, even more, profound awe perceptions that require an adjustment of frame of reference, we found hints of second-order experiences in terms of *wondering* and also immediate first-order experiences of *awe*, as differentiated by Gallagher et al. (2015). Several new insights emerged in retrospect, as the consequence of RBSs´ reflective processes triggered by experiences; these could be regarded as spiritual changes or transformations. Some experiences can change a person’s life, as they are so significant that they open up a new perspective on reality, resulting in an alternative way of life (Bill, 2019). However, not all experiences are that profound, yet even the small moments of amazement lift people (for a moment) out of the ordinary and inspire them.

The description of triggers may encourage others to be mindfully aware of similar situations in their life, thereby enriching their life by pausing in specific moments of attraction, fascination, wondering awe, and reflection.

## Data Availability

Not applicable.
